# Multifocal tuberculosis simulating a cancer-a case report

**DOI:** 10.1186/s12879-020-05209-x

**Published:** 2020-07-10

**Authors:** Congxiao Wang, Ying Luan, Shifeng Liu, Mingwei Zhao, Hao Zhang, Wei Li, Zixiang Li, Xiaokun Hu, Lijing Peng

**Affiliations:** 1 Department of the Interventional Medical Center, The Affiliated Hospital of Qingdao University, Qingdao, 266000 Shandong China; 2 Jiangsu Key Laboratory of Molecular and Functional Imaging, Department of Radiology, Zhongda Hospital, Medical School of Southeast University, Nanjing, 210009 China; 3 Qingdao Chest Hospital, Qingdao, 266000 Shandong China; 4 Department of Clinical Laboratory, The Affiliated Hospital of Qingdao University, Qingdao, 266000 Shandong China

**Keywords:** Case report, Multifocal tuberculosis, System malignancy, PET/CT, Biopsy

## Abstract

**Background:**

Tuberculosis is a disease that may affect any organ of the body. Multifocal tuberculosis involving multiple systems with associated symptoms are rare, which makes the diagnosis challenging. Distinguishing multifocal tuberculosis from lesions metastatic from system malignancy is difficult. Single detection method is difficult to make a diagnosis. A combination of multiple methods is essential.

**Case presentation:**

A 17-year-old male presented with a 20 days weakness of lower limbs, which aggravated for 6 days. The PET/CT showed increased metabolism of ileocecal intestinal and terminal ileum wall, multiple enlarged lymph node (LNs), multiple osteolytic bone lesions, and soft tissue intensity belong T7 and T8 vertebrae. To confirm the diagnosis of the disease, a biopsy of the mediastinum lymph nodes was carried out. Polymerase chain reaction (PCR) test of the specimen was positive for the *Mycobacterium tuberculosis*, the T-SPOT and Xpert MTB/RIF test were also positive, which suggested the presence of *Mycobacterium tuberculosis*. The final diagnosis was multifocal tuberculosis, the patients received the resection of the mass in the spine. Anti-tuberculosis drugs were given. The myodynamia and muscle tension of the patients recovered following the therapy.

**Conclusions:**

Our results indicated that Multifocal tuberculosis should also be taken into consideration when lesions metastatic from system malignancy were suspected from images results even without the clinical symptoms of tuberculosis, and combination of multiple diagnosis methods were essential for the diagnosis of multifocal disease.

## Background

As an infectious disease among adults, tuberculosis (TB) is the leading cause of death worldwide [1]. As WHO reported, there were approximately 10 million new cases of TB in 2017, among which only 6.4 million were diagnosed. Approximate 1.3 million people die due to TB each year [2, 3]. Although the diagnosis of TB had made multiple advances in recent years, diagnosis of TB with reliable, simple, point-of-care test is still necessary. Clinicians often depend on the bacteriological diagnosis, but clinical findings, radiological evidence, and tests for bacterial products are also important clues for the presence of M. tuberculosis [2]. Multifocal tuberculosis involving multiple systems with associated symptoms are rare. Distinguishing multifocal tuberculosis from lesions metastatic from system malignancy is difficult. A range of diagnostic and drug susceptibility test was essential for the final diagnosis. Here, we reported a case of multifocal tuberculosis diagnosed with combination of multiple methods.

## Case presentation

A 17-year-old male presented with a 20 days weakness of lower limbs, which aggravated for 6 days. He had a cough accompanied by a small amount of white sputum, while without fever, hemoptysis, respiratory stress, chest pain, abdominal pain, diarrhea, bloating, headache, dizziness or other specific symptoms. The sputum was collected for analysis, but the result was negative.

**Fig. 1:**
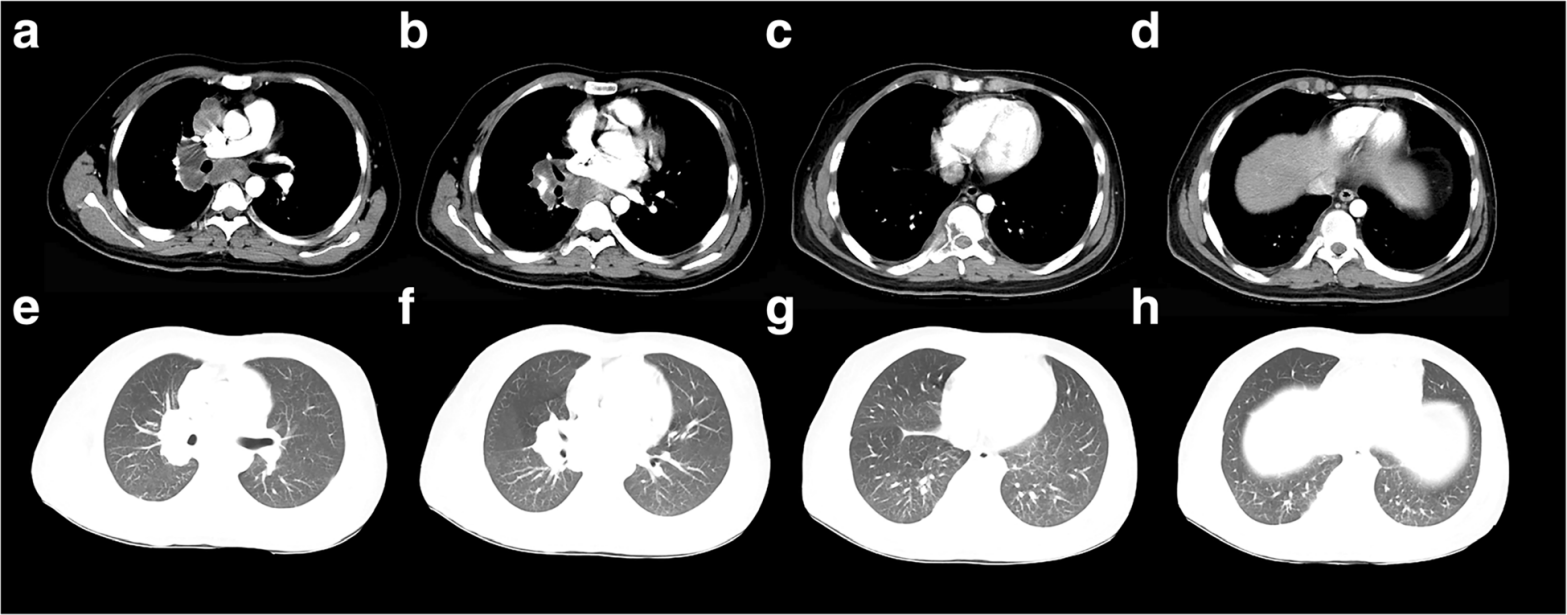
Representative slices of enhanced chest CT images showed **a**, **b** multiple enlarged lymph nodes in the mediastinal and right hilus of lung, **c**, **d** bone destruction and soft tissue density mass in the body and appendage of T8, and right eighth rib head and **e**-**h** chronic inflammation changes of the lungs

**Fig. 2:**
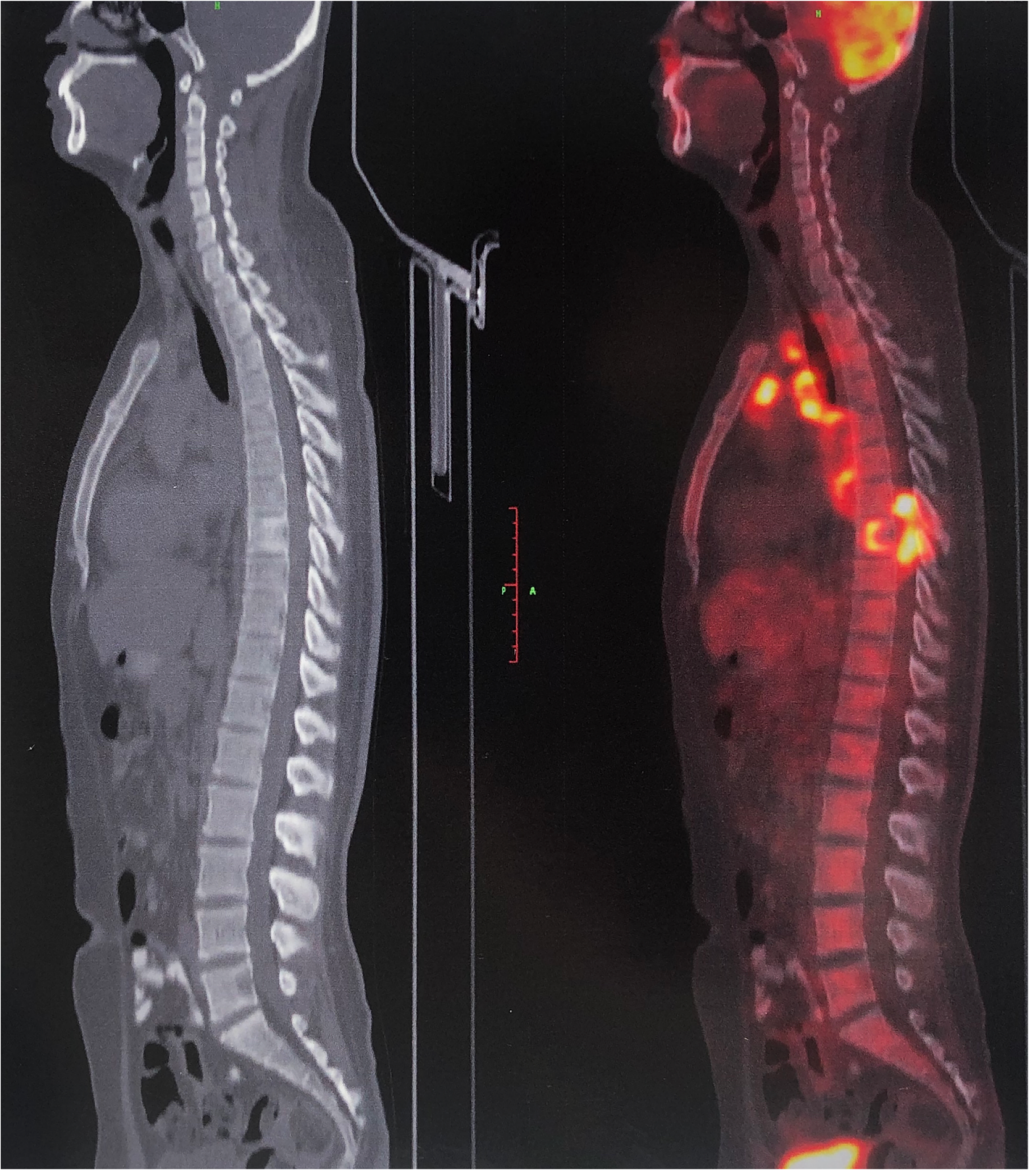
Maximum intensity projection image of PET/CT showing the high metastasis in ileocecal intestinal and terminal ileum, multiple LNs, bones and soft tissue

**Fig. 3:**
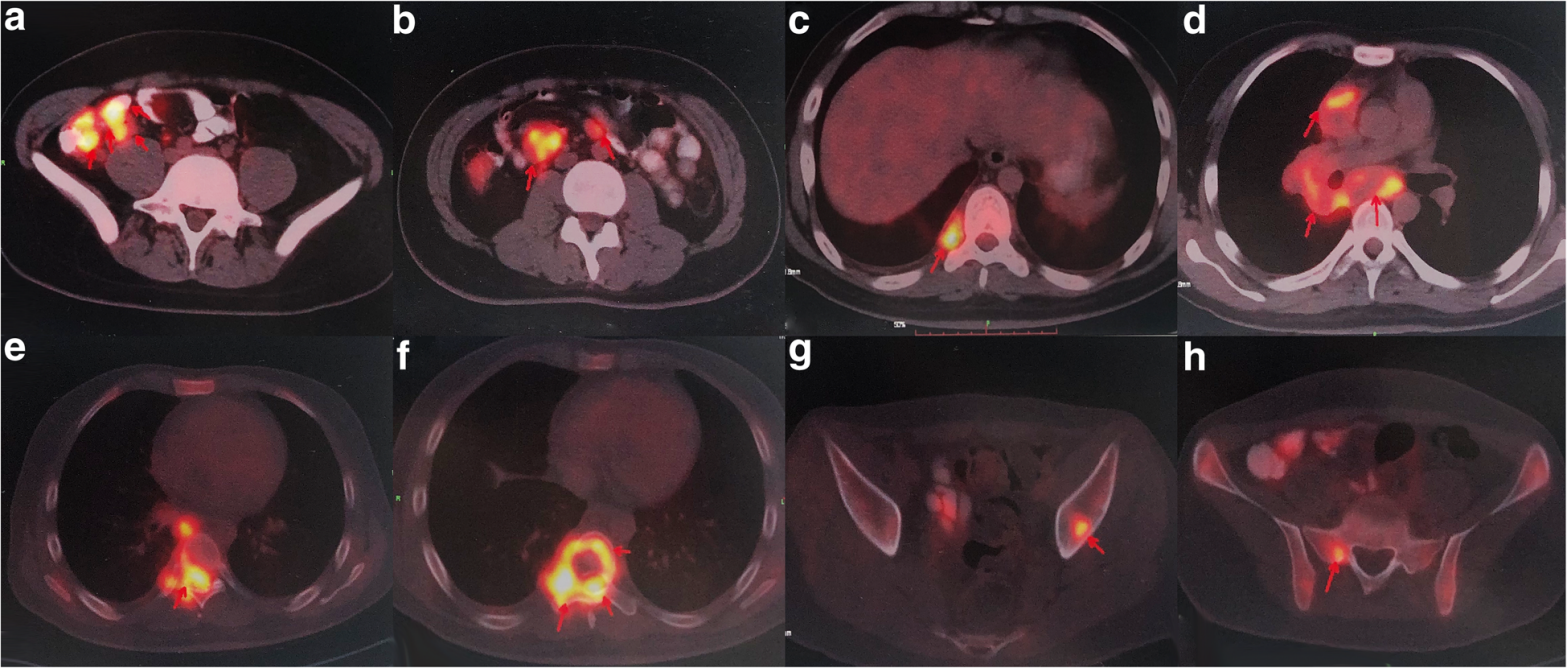
Selected transaxial slices of high metastasis in the mass in the **a** ileocecal intestinal and terminal ileum wall, **b**-**d** multiple LNs, and **e**-**h** bones

His medical history included no hepatitis, TB, malaria, hypertension, diabetes mellitus, heart disease, cerebrovascular disease, psychosis, operation, trauma, blood transfusion, allergies. The patients declared no exposure history to active TB in the past. His heart rate was 112 beats per minute, blood pressure 103/77 mmHg, respiratory rate 25 breaths per minute, temperature 36.3 °C, height 182 cm, weight 66.5 kg. The myodynamia of the right lower limb decreased to grade 3 with a decreased muscle tension. The myodynamia of the left lower limb decreased to grade 4, while the muscle tension was normal. Pre-admission enhanced chest CT showed that chronic inflammation changes in both lungs were found. Multiple enlarged lymph nodes (LNs) were observed in the mediastinal and right hilus of lung. Bone destruction and soft tissue density mass were found in the body and appendage of T8, and right eighth rib head, metastatic tumor was considered (Fig. 1). PET/CT showed thickening of the ileocecal intestinal and terminal ileum wall, increasing metabolism with SUVmax approximate 12.0 and 9.7 respectively. Multiple enlarged lymph nodes (LNs) were observed, partial of which necrosis occurred, with SUVmax approximate 15.3. PET/CT showed multiple osteolytic bone lesions, including the appendage of T7, the body and appendage of T8, right eighth rib head, parapophysis of T11, body of S1 and left ilium, soft tissue intensity was observed belong T7 and T8, protruding into the spinal canal, with SUVmax approximate 13.5. The PET/CT results suggested the following diagnosis: Cancer of terminal ileum and ileocecal ileum, with multiple metastasis including LNs and bone; multiple lymphomas; multiple tuberculosis (Fig. 2, Fig. 3).

The blood routine tests showed increased white blood cell (9.98 × 10^9/L), neutrophils (7.09 × 10^9/L), monocytes (0.89 × 10^9/L), and blood platelet (430 × 10^9/L). C-reactive protein was also at a high level (24.05 mg/L). The red blood cell was 4.65 × 10^12/L and the hemoglobin was 130 g/L. Routine coagulation tests showed increased fibrinogen (4.06 g/L) and D-dimer (3130 ng/ml), the prothrombin time (11.2 s), activated partial thromboplastin time (22.8 s) and international normalized ratio (0.96) were in the normal range. Liver function (albumin: 40.1 g/L, Tbil: 14.3 umol/L, direct bilirubin: 5.3 umol/L, indirect bilirubin: 9 umol/L, alanine transaminase: 19.2 U/L, aspartate transaminase: 25.9 U/L), renal function (urea: 2.63 mmol/L, creatinine: 359 umol/L), electrolytes (potassium: 4.8 mmol/L, sodium: 136.7 mmol/L, chlorine: 99.8 mmol/L), urine routine tests (urine protein: +−)**,** stool routine tests (fecal occult blood: weak positive) provided no valuable diagnostic information. Anti-human immune deficiency virus (HIV) antibodies, treponema pallidum antibodies, hepatitis B surface antigen (HbsAg), and hepatitis C virus antigen (HcvAg) were all negative. Neuronspecific enolase was 26.51 ng/ml, while the other related male tumor makers were within a normal range (Table 1).

**Table 1:** Laboratory exams of the patient

Characteristics	Values	Reference Range
**Blood routine tests**		
White blood cell (× 10^9/L)	9.98	3.5–9.5
Neutrophils (× 10^9/L)	7.09	1.8–6.3
Monocytes (× 10^9/L)	0.89	0.1–0.6
Red blood cell (× 10^12/L)	4.65	4.3–5.8
Hemoglobin (g/L)	130	130–175
Blood platelet (× 10^9/L)	430	100–300
C-reactive protein (mg/L)	24.05	0–5
**Routine coagulation tests**		
PT (sec)	11.2	10–14
APTT (sec)	22.8	22–38
INR	0.96	0.8–1.2
Fibrinogen (g/L)	4.06	2–4
D-dimer (ng/ml)	3130	0–500
**Liver function**		
Albumin (g/L)	40.1	35–55
Tbil (umol/L)	14.3	3–22
Direct bilirubin (umol/L)	5.3	0–8
Indirect bilirubin	9	
ALT (U/L)	19.2	9–50
AST (U/L)	25.9	15–40
**Renal function**		
Urea (mmol/L)	2.63	3.1–8.0
Creatinine (umol/l)	359	31–132
**Electrolytes**		
Potassium (mmol/L)	4.8	3.5–5.3
Sodium (mmol/L)	136.7	137–147
Chlorine (mmol/L)	99.8	99–110
**Stool routine tests**		
Fecal occult blood	weak positive	negative
**Urine routine tests**		
Urine protein	+−	negative
**Cancer marker**		
NSE (ng/ml)	26.51	0–17
**T-SPOT**		
T-SPOT	+	negative

Note.- *PT* prothrombin time, *APTT* activated partial thromboplastin time, *INR* International Normalized Ratio, *Tbil* total bilirubin, *ALT* alanine transaminase, *AST* aspartate transaminase, *NSE* Neuronspecific enolase

**Fig. 4:**
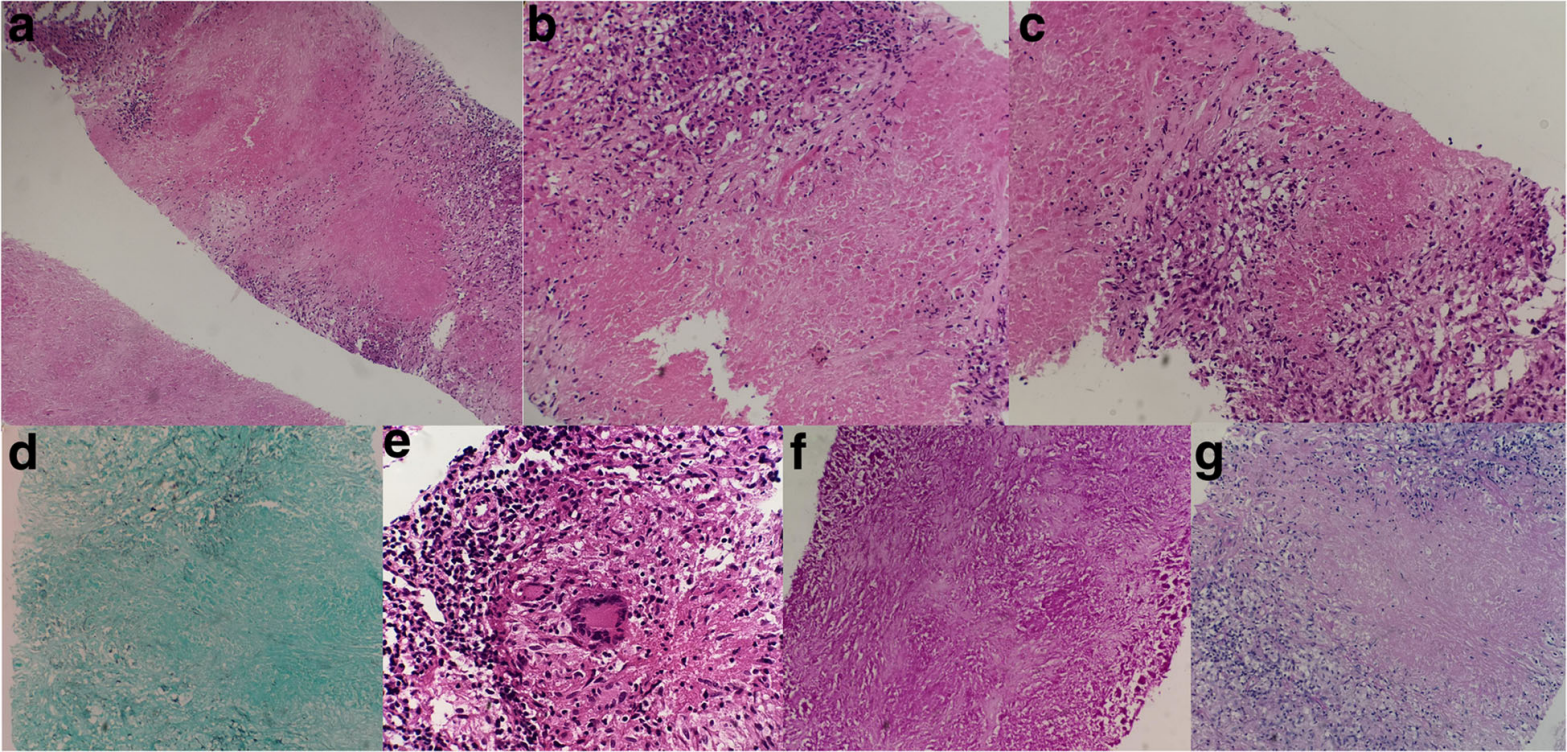
Histology results after biopsy. **a**-**c** H&E staining showed granulomas and caseous necrosis. **d** AFB **e** PAS **f** GMS and **g** gram staining showed no positive results

To confirm the diagnosis of the disease, a biopsy of the mediastinum lymph nodes was carried out. Histopathological examination revealed granulomas and caseous necrosis, which was consistent with TB, while AFB (Acid Fast Bacteria), PAS (Periodic Acid-Schiff Stain), GMS (Grocott Methenamine Silver Stain) and gram staining showed no positive results (Fig. 4). Polymerase chain reaction (PCR) test and Xpert MTB/RIF test of the specimen, and the blood T-SPOT test were also positive (Table 1), which suggested the presence of *Mycobacterium tuberculosis*. The final diagnosis was multifocal tuberculosis. Mass in the spine caused progressively aggravated lower limb weakness over time, the myodynamia of the patient decreased to grade 2, thus resection of the mass in the spine was carried out. Eight weeks after the resection, the myodynamia of the patient recovered to grade 5. No positive results were found 6 weeks after the bacterial culture, and Xpert MTB/RIF results showed a positive result of the *Mycobacterium tuberculosis* gene, but no rifampicin resistance gene mutation was found. Therefore, we treated the patient as the drug-susceptible tuberculosis. The patient was given a daily oral anti-tuberculosis drug therapy consisting of isoniazid (300 mg/d), rifampicin (600 mg/d), pyrazinamide (1500 mg/d), ethambutol hydrochloride (1000 mg/d), mecobalamin (1.5 mg/d). The therapy with pyrazinamide will last for 6 months, the mecobalamin was discontinued after 2 months when the myodynamia recovered, and the other medications were recommended to 18 months. At the time of last follow up, the patient regained his normal activity with the myodynamia recovered to grade 5, no obvious abnormalities were found in the laboratory examination, the imaging examination showed obvious reduction in the occupation.

## Discussion and conclusions

TB is one of the most popular infectious diseases in China, each year, around 250,000 people died due to TB in China [4]. However, some of the patients with TB are hard to diagnosis, especially for those that mimic the cancers [5, 6].

As showed in this case, the clinical manifestation and laboratory test showed no valuable clues for the diagnosis of TB at the time of admission. Radiological screening gives important clues for the exits of TB, and also WHO recommended it as an important assessment method for the diagnosis of TB [2]. PET/CT can provide metabolic differences between normal cells and malignant cells. However, false-positive cases are constantly reported, as the F-18 FDG could be absorbed not only by tumor cells but also by inflammatory or infective lesions [7, 8]. Although various imaging examinations are available for the diagnosis of different kinds of disease. Differentiation of multifocal tuberculosis with a malignancy is difficult. In the present cases, the PET/CT results showed multifocal high metabolism. The multifocal tuberculosis with the multifocal high metabolism showed on PET/CT is easy to mistake for a malignancy, especially in this case, the patients showed occupation in the vertebrae, and the myodynamia and muscle tension were affected. Studies have showed that LNs are the second most common sites of TB infection [9], and also one of the most common sites for the metastasis of a malignancy. That’s why we chose LNs as the site for the biopsy. In this case, the results of the biopsy suggested TB, however, positive results of TB depend on the load of bacterial in the tissue. Thus, negative results could not exclude the existence of TB. In this situation, combination of multiple methods for the diagnosis is necessary.

The histopathology indicated a TB, and also the PCR test of the specimen, the blood test of T-SPOT and the Xpert MTB/RIF results suggested the exist of *Mycobacterium tuberculosis*. The result suggested the importance of combination of multi-methods for the diagnosis of disease. Surgery combined with anti-tuberculosis and neurotrophic medications promoted the recovery of the patient.

The present case indicated that multifocal tuberculosis should also be taken into consideration when lesions metastatic from system malignancy were suspected from images results even without the clinical symptoms of TB, and combination of multiple diagnosis methods were essential for the diagnosis of multifocal disease.

## Data Availability

The datasets used and/or analysed during the current study are available from the corresponding author on reasonable request.

## Abbreviations

LNs: Lymph node
PCR: Polymerase chain reaction
HIV: Anti-human immune deficiency virus
HbsAg: Hepatitis B surface antigen
HcvAg: Hepatitis C virus antig
TB: Tuberculosis
AFB: Acid Fast Bacteri
PAS: Periodic Acid-Schiff Stain
GMS: Grocott Methenamine Silver Stain
